# Restructuring of *Enterococcus faecalis* biofilm architecture in response to antibiotic-induced stress

**DOI:** 10.1038/s41522-017-0023-4

**Published:** 2017-06-30

**Authors:** Jennifer L. Dale, Jennifer L. Nilson, Aaron M. T. Barnes, Gary M. Dunny

**Affiliations:** 1 Department of Microbiology and Immunology, University of Minnesota, Minneapolis, MN USA; 2 Department of Lab Medicine and Pathology, University of Minnesota, Minneapolis, MN USA

## Abstract

Bacterial biofilms are intrinsically resistant to antimicrobial treatment, which contributes to microbial persistence in clinical infections. *Enterococcus faecalis* is an opportunistic pathogen that readily forms biofilms and is the most prevalent enterococcal species identified in healthcare-associated infections. Since intrinsic resistance to multiple antibiotics is common for enterococci, and antibiotic resistance is elevated in biofilm populations, it is imperative to understand the mechanisms involved. Previously, we identified two glycosyltransferase genes whose disruption resulted in impaired nascent biofilm formation in the presence of antibiotic concentrations subinhibitory for parent growth and biofilm formation. The glycosyltransferases are involved in synthesis of the cell-wall-associated rhamnopolysaccharide Epa. Here we examined the effect of *epa* mutations on the temporal development of *E. faecalis* biofilms, and on the effects of antibiotics on pre-formed biofilms using scanning electron microscopy. We show that Δ*epaOX* mutant cells arrange into complex multidimensional biofilms independent of antibiotic exposure, while parent cells form biofilms that are monolayers in the absence of antibiotics. Remarkably, upon exposure to antibiotics parent biofilm cells restructure into complex three-dimensional biofilms resembling those of the Δ*epaOX* mutant without antibiotics. All biofilms exhibiting complex cellular architectures were less structurally stable than monolayer biofilms, with the biofilm cells exhibiting increased detachment. Our results indicate that *E. faecalis* biofilms restructure in response to cellular stress whether induced by antibiotics in the case of parent cells, or by deficiencies in Epa composition for the Δ*epaOX* strain. The data demonstrate a link between cellular architecture and antibiotic resistance of *E. faecalis* biofilms.

## Biofilm formation: Architecture and antibiotics

Studying how the architecture of bacterial biofilms can change on exposure to antibiotics could help tackle persistent clinical infections. *Enterococcus faecalis* is one of the most prevalent bacteria involved in healthcare situations. Its resistance to antibiotics is elevated by the formation of biofilms. Gary Dunny and colleagues at the University of Minnesota, USA studied genetic factors related to the response of *E. faecalis* biofilms to antibiotics. They identified mutations that caused the bacterial cells to produce structurally altered biofilms and found that similar structural alterations were induced in biofilms made by normal cells in the presence of antibiotics. The altered architecture can make the biofilms less stable and therefore more easily disrupted by treatments. Learning more about the mechanisms underlying these structural changes in biofilms might lead to new options in treating antibiotic resistant infections.

## Introduction

Antibiotic resistance is a significant burden in healthcare-associated infections (HAIs),^1^ and is exacerbated by the presence of biofilms. It is estimated that biofilm populations are 10–1000 times more resistant to antibiotics than planktonic cells.^2, 3^ Several diverse mechanisms have been implicated in enhanced biofilm resistance to antibiotics including decreased antibiotic penetration, sequestration of antibiotics, and the presence of persister cells.^4–6^

Enterococci are among the most common pathogens found in HAIs with *Enterococcus faecalis* being the primary enterococcal species identified.^1^ *E. faecalis* readily forms biofilms on a wide-range of natural and artificial substrates including indwelling medical devices, damaged heart valves, and catheters, which contributes to its pathogenicity.^7, 8^ We identified recently two glycosyltransferase (GTF) genes that contribute to *E. faecalis* biofilm-associated antibiotic resistance. Deletion of either GTF *epaI* or *epaOX* impaired nascent biofilm formation in the presence of subinhibitory concentrations of antibiotics, with negligible reduction of biofilm formation in the absence of antibiotics.^9^ The GTFs are involved in the synthesis of a cell-wall-associated polysaccharide and are linked to the enterococcal polysaccharide antigen (*epa*) gene cluster.

Various functions have been described for *epa* genes in *E. faecalis*. Certain *epa* genes are important determinants of Epa polysaccharide composition, biofilm formation, phage susceptibility, and cellular morphology.^9–12^ Deletion of *epa* genes resulted in decreased virulence in murine peritonitis and ascending urinary tract infection models,^13, 14^ impaired infectivity in *Galleria mellonella*,^15^ and diminished murine intestinal colonization.^10^ Our lab and others have shown that deletion of various *epa* genes decreased cell-wall integrity and increased cell envelope permeability.^9, 16^ Recently, Hoff et al.^16^ demonstrated that defects in Epa polysaccharide production increased susceptibility of *E. faecalis* planktonic cells toward cephalosporin antibiotics. While the phenotypical consequences of Epa alterations continue to expand, our understanding of Epa structure and how it contributes to virulence and intrinsic antibiotic resistance remains incomplete.

In this study, we sought to determine how *epaI* and *epaOX* enhance antibiotic resistance in *E. faecalis* during biofilm growth. Here we show that deletion of *epaOX* results in the most noticeable phenotypic differences in biofilm architecture and biochemical composition. We also provide evidence that exposure of parent biofilms to subinhibitory concentrations of antibiotics results in restructuring of biofilms into a complex three-dimensional (3D) architecture reminiscent of Δ*epaOX* biofilms grown in the absence of antibiotics. In addition, the structural stability of *E. faecalis* biofilms exhibiting increased 3D cellular architecture is significantly diminished relative to monolayer biofilms, even though biofilm biomass is not reduced. We propose that formation of a complex 3D biofilm structure in the presence of antibiotics, or as a result of cell-wall alterations, may constitute a stress response of enterococcal biofilm cells.

## Results

### *epaI* and *epaOX* gene expression is unaffected by biofilm growth

We reported previously that the GTFs *epaI* and *epaOX* contributed to biofilm-associated antibiotic resistance using standard microtiter assays quantifying stained biofilm biomass as a measurement of biofilm growth. Deletion of either GTF resulted in wild-type biofilm formation in the absence of antibiotic and decreased nascent biofilm formation in the presence of subinhibitory concentrations of clinically relevant antibiotics.^9^ To initiate studies identifying the mechanism of biofilm-associated antibiotic resistance related to the *epa* genes, we considered whether there were differences in *epaI* and *epaOX* gene expression in biofilm vs. planktonic populations. We hypothesized that if the *epa* genes were upregulated in biofilm cells, this could increase synthesis of the Epa polysaccharide and increase resistance to antibiotics. The results of quantitative reverse transcription PCR (RT-qPCR) revealed the relative fold-change in gene expression of *epaI* (0.9 +/− 0.042) and *epaOX* (1.0 +/− 0.002) was unchanged between biofilm and planktonic cell populations. These data indicate that differential *epa* gene expression in planktonic and biofilm populations is not responsible for biofilm-associated antibiotic resistance.

### Biofilm architecture is impacted by glycosyltransferases

**Fig. 1:**
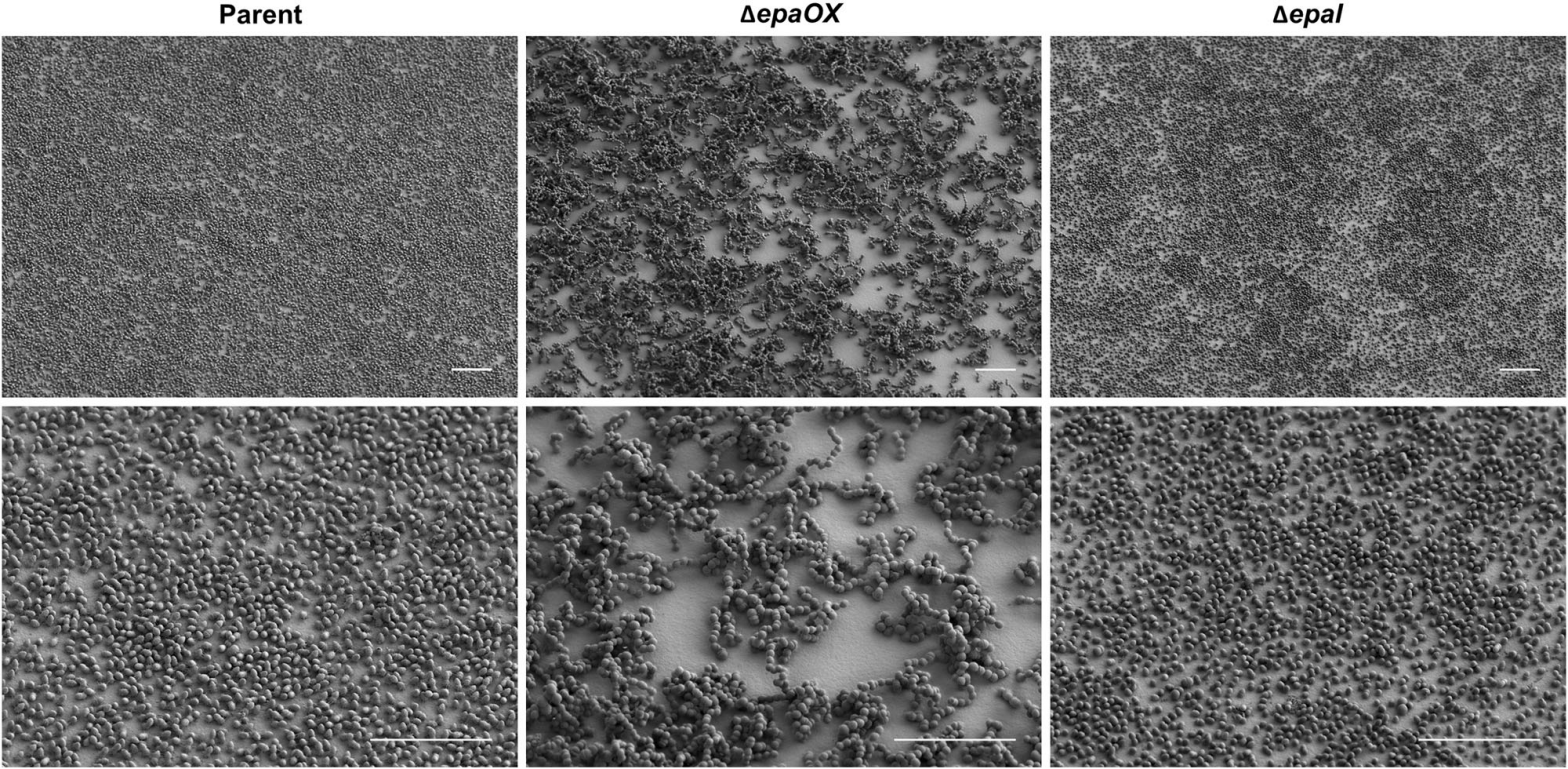
Effects of *epa* deletions on *E. faecalis* biofilm formation. Scanning electron micrographs showing structural differences in biofilm architecture of parent strain OG1RF vs. isogenic *epa* mutants. Cultures were grown on Aclar membranes in CBRs for 24 h in the absence of antibiotics (scale bars, 10 µm). Representative images are shown from experiments performed a minimum of three times using biological replicates

We sought to examine the effect of *epa* mutations on the architecture of *E. faecalis* biofilms considering that Epa cell-wall polysaccharides may affect surface properties, and may also contribute to the biofilm extracellular matrix. Parent and *epa* mutant strains were cultured on Aclar fluropolymer membranes in a continuous flow CDC biofilm reactor (CBR) for 24 h and biofilm architecture was assessed using scanning electron microscopy (SEM) (Fig. 1). The parent strain formed a monolayer biofilm, occasionally producing small microcolonies approximately two cells in depth. Deletion of *epaOX* resulted in production of a markedly different biofilm architecture. The Δ*epaOX* cells chained and clumped resulting in a complex 3D biofilm that was unable to completely cover the surface area of the Aclar membrane, which led to areas devoid of attached bacteria. Biofilms of the *epaI* mutant were more similar in appearance to parent. Since the Δ*epaOX* strain showed the most dramatic changes in biofilm architecture, we decided to examine further the differences between parent and Δ*epaOX* biofilms and the effects of antibiotics on biofilm structure.

### *E. faecalis* restructures into microcolony biofilms in response to antibiotics

**Fig. 2:**
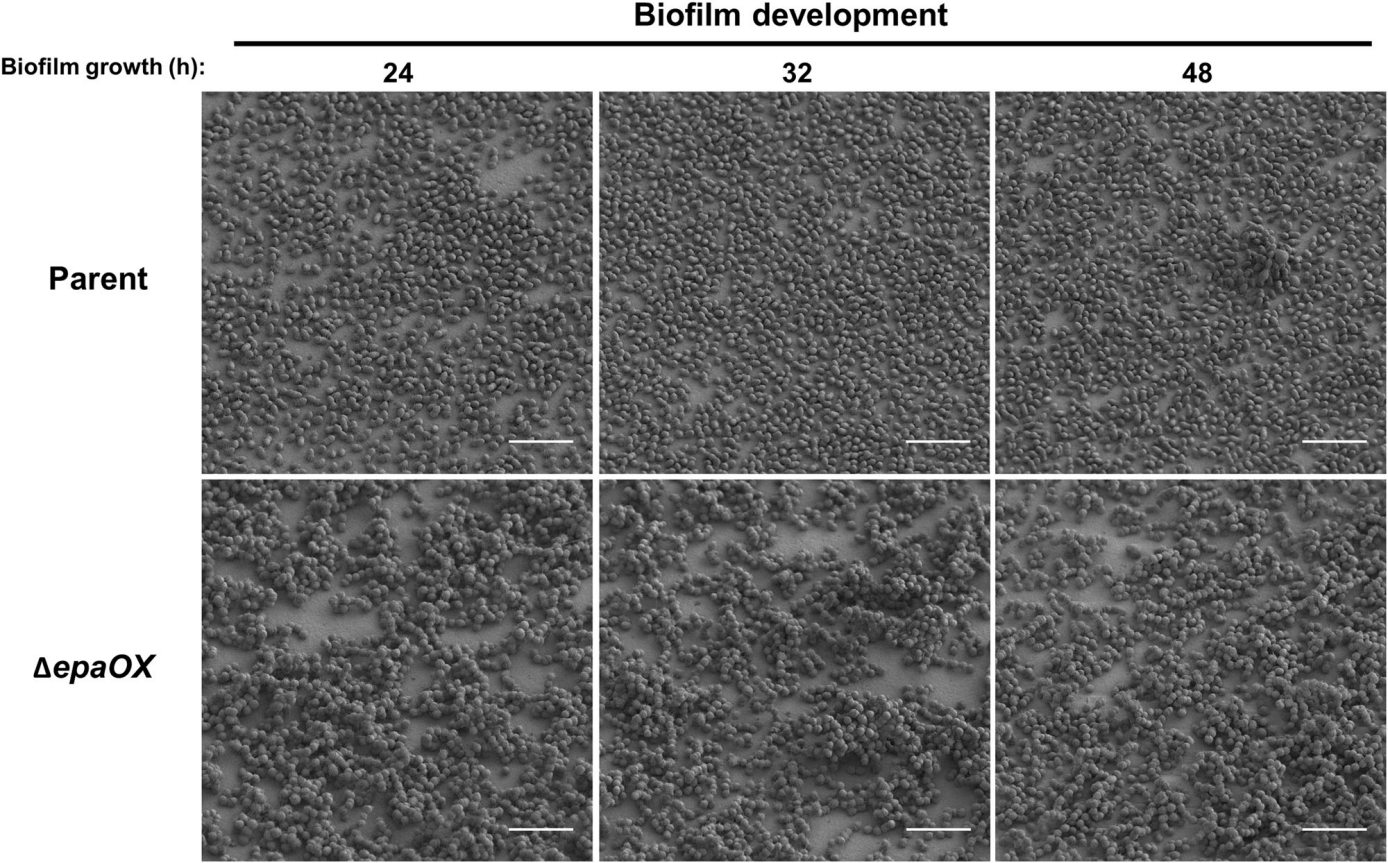
Biofilm development of *E. faecalis* parent and Δ*epaOX* strains after extended cultivation. Experiments were performed as detailed in Materials and Methods. *E. faecalis* biofilms were cultured on Aclar membranes in CBRs for either 24, 32, or 48 h without antibiotic prior to fixation. The biofilms from each time point were visualized using low-voltage scanning electron microscopy to examine biofilm architecture (scale bars, 5 µm). Representative images are shown from experiments performed in triplicate with similar results

To determine the impact of antibiotics on established biofilms, we cultured parent and Δ*epaOX* strains in CBRs for 24 h without antibiotic and then exposed the resulting biofilms to antibiotic and examined architecture using SEM. The antibiotics used were chosen based on clinical relevance, and the concentrations were chosen based on previous results demonstrating that these concentrations were subinhibitory for planktonic growth.^9^ In the absence of antibiotics, parent cells formed monolayer biofilms that resulted in the formation of occasional microcolonies greater than two cells in depth after 48 h of growth. SEM micrographs showed that the biofilms of the Δ*epaOX* mutant remained the same throughout the 48 h time course with cells chaining, clumping, and forming complex 3D biofilms (Fig. 2). Both parent and Δ*epaOX* biofilm cells appeared to form cellular appendages, or protrusions, that extended from the cells. The appendages remained over the time course of the experiment with subtle decreases in both strain backgrounds at 48 h. In addition, parent and Δ*epaOX* strains remained viable over the 48 h time course in the absence of antibiotic as observed using the immunofluorescence stain Syto9. Few cells stained with propidium iodide (PI), which stains dead cells or cells with increased permeability (Supplementary Fig. 1).

**Fig. 3:**
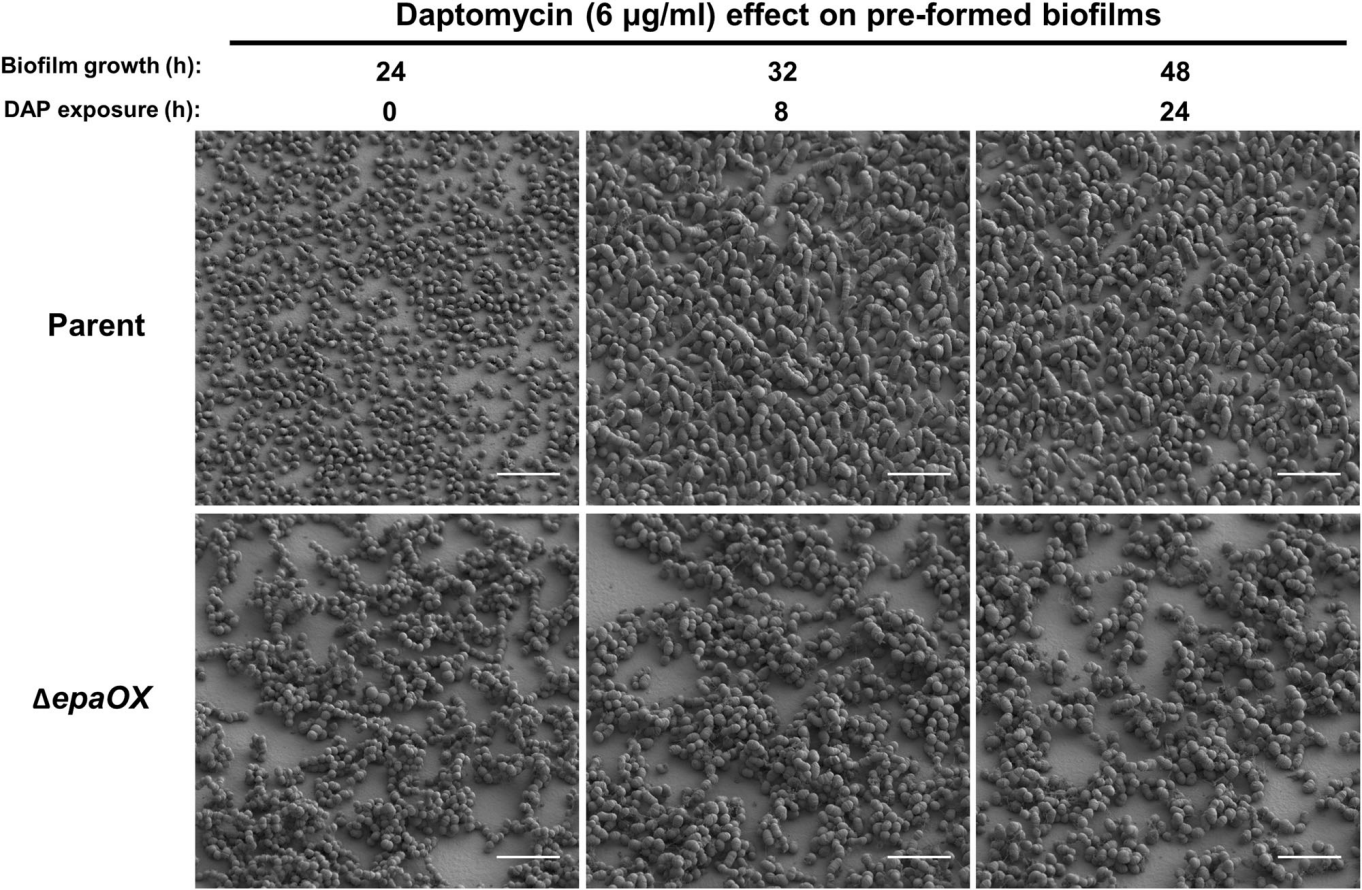
Daptomycin effects on pre-formed *E. faecalis* biofilms. Experiments were performed as detailed in Materials and Methods. *E. faecalis* parent and Δ*epaOX* strains were cultured on Aclar membranes for 24 h in CBRs without antibiotics to establish biofilms. Daptomycin (DAP) was then added to the culture medium at a concentration of 6 µg/ml and the effects on the pre-formed biofilms were assessed after 8 and 24 h of antibiotic exposure. Low-voltage scanning electron microscopy (SEM) was performed to examine biofilm architecture (scale bars, 5 µm). Representative images are shown from experiments performed in triplicate with similar results

**Fig. 4:**
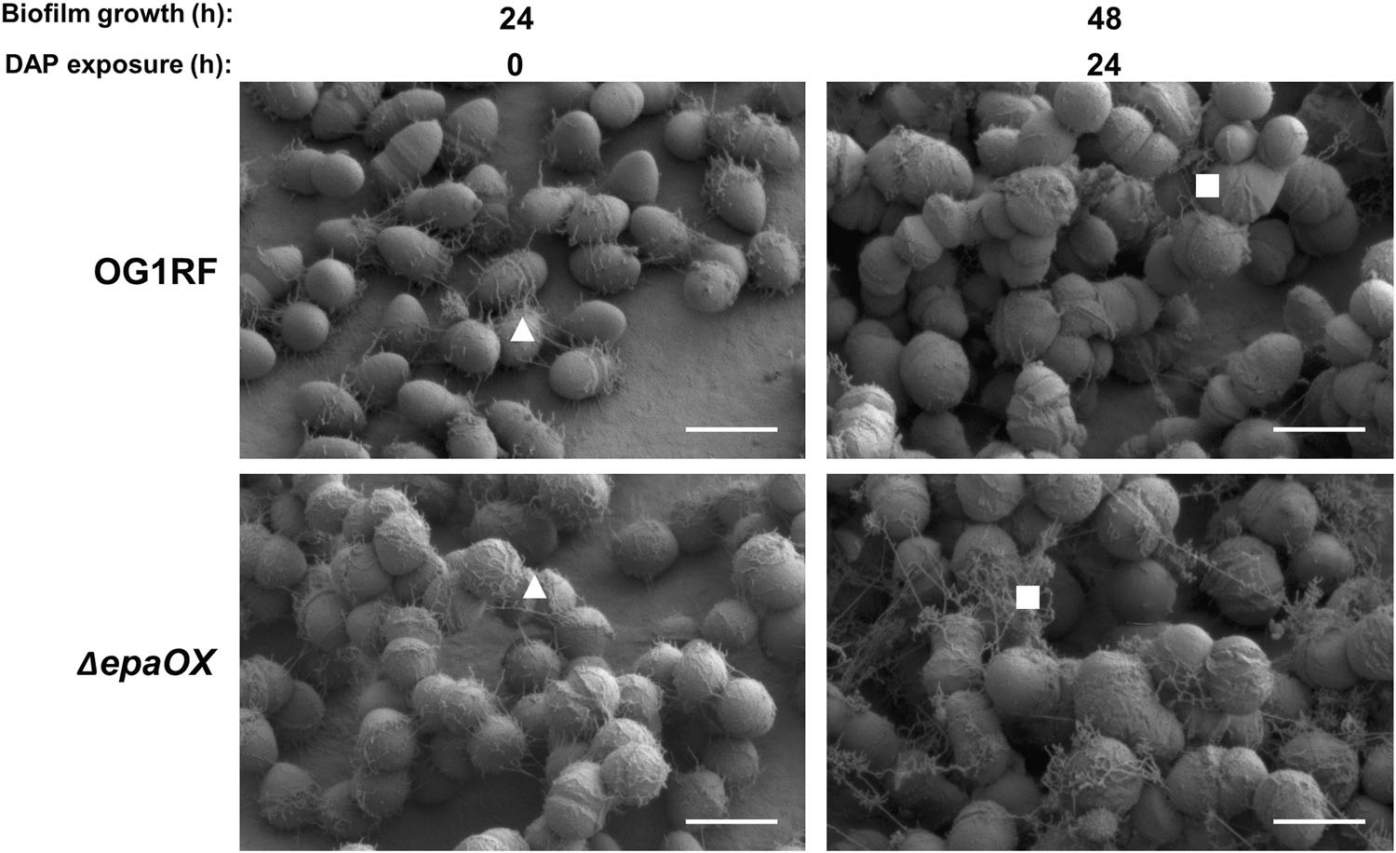
Daptomycin exposed biofilms release extracellular material. Low-voltage scanning electron microscopy (SEM) demonstrating the presence of cellular appendages (*triangles*) produced by *E. faecalis* biofilms not exposed to antibiotics compared to extracellular debris (*squares*) released from biofilms exposed to daptomycin (DAP) (scale bars, 1 µm)

The most dramatic morphological changes in biofilm architecture appeared after parent biofilms were exposed to subinhibitory concentrations of antibiotic (Fig. 3 and Supplementary Fig. 2). After 8 h exposure to daptomycin (DAP), parent biofilm architecture changed drastically from monolayers into complex 3D architectures whereas Δ*epaOX* biofilm architecture remained unchanged upon exposure to DAP. In addition, cell division of both parent and Δ*epaOX* strains was affected with the most noticeable antibiotic-induced changes in cellular morphology occurring in the parent strain. DAP has been shown previously to affect cell division in *E. faecalis* and other bacterial species.^17–19^ Live/dead staining for both strains indicated increased uptake of PI at 8 and 24 h exposure to DAP suggestive of cell death (Supplementary Fig. 1). However, PI has the ability to also stain cells with increased permeability and extracellular DNA. Higher magnification SEM micrographs demonstrated the presence of extracellular material, which was visually distinct from the previous observation of cellular appendages, in the parent and Δ*epaOX* strains (Fig. 4). These results suggest there is increased cell lysis and/or release of intracellular content from biofilm cells exposed to DAP, with somewhat higher levels of released material evident in the Δ*epaOX* biofilms (Fig. 4). Similar, albeit more subtle, restructuring of the parent biofilm occurred after exposure to gentamicin (Gm) (Supplementary Fig. 2). After 8 h exposure to Gm, parent cells started clustering into small microcolonies that were more apparent by 24 h. The Δ*epaOX* mutant continued to form the same complex 3D architecture biofilms in the presence of Gm as was observed when cultured without antibiotics. There was no significant PI staining until parent and Δ*epaOX* mutants had been exposed to Gm for 24 h (Supplementary Fig. 1). Higher magnification SEM micrographs further demonstrated microcolony formation and increases in regions of the surface devoid of adherent bacteria after Gm exposure in the parent strain. Interestingly, both parent and Δ*epaOX* cells were devoid of cellular appendages when exposed to Gm (Supplementary Fig. 2b). In addition, Δ*epaOX* biofilms were susceptible to both DAP and Gm exposure as measured using quantitative counts indicating that both antibiotics were capable of interacting with the biofilm cells even though no perceptible architectural changes were observed (Supplementary Fig. 3). Since the parent biofilm restructuring could be explained by the attachment of planktonic cells into nascent microcolonies, we examined planktonic cell expansion in microtiter growth curve assays. Using culture medium identical to the CBR experiments (10% TSB^-d^ with or without antibiotics), there was no significant increase in the OD_600_ of parent or Δ*epaOX* cells indicating no cell growth/expansion (data not shown). In total, these data suggest that parent restructuring from monolayers into microcolonies is mediated by the division or rearrangement of bacterial cells attached prior to antibiotic treatment vs. new colonization by planktonic cells in the CBR vessel.

### Microcolony biofilms show reduced structural integrity relative to monolayer biofilms

**Fig. 5:**
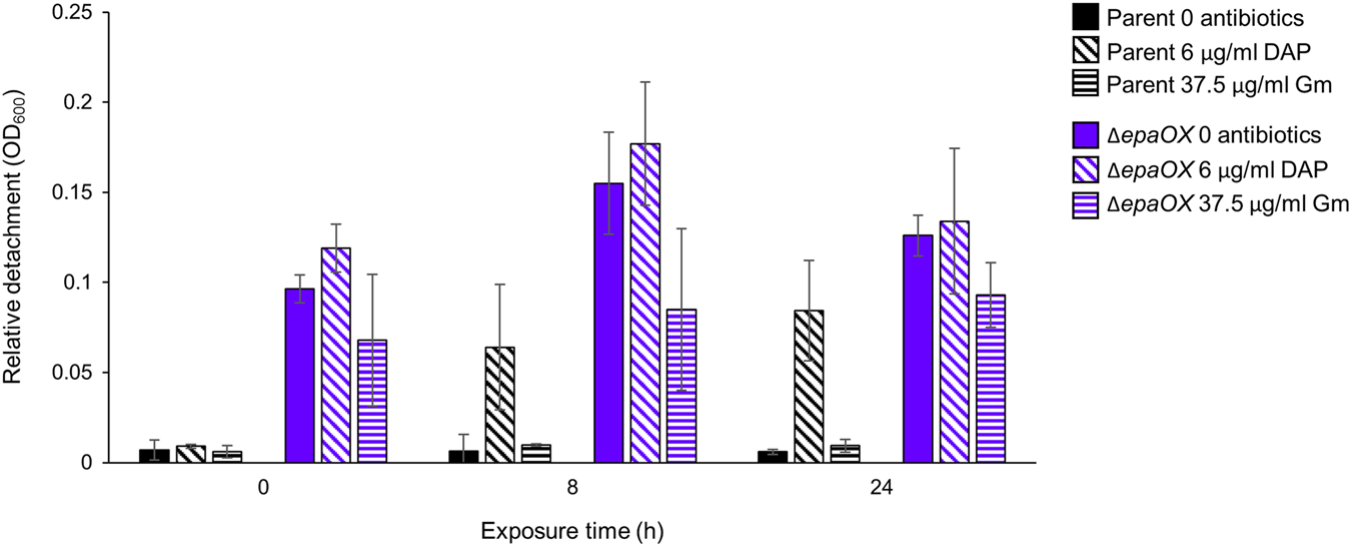
*E. faecalis* biofilm stability. Relative detachment of biofilms cells from Aclar membranes after washing, as determined by measuring the OD_600_ of the wash buffer as described in the methods. Error bars represent standard deviation (*n* = 2 for 0 antibiotics; *n* = 3 for DAP and Gm)

While removing samples from the CBRs for imaging, we observed turbidity differences between parent and Δ*epaOX* in the liquid phase of the CBR cultures following antibiotic exposure. This suggested that the structural stability of *E. faecalis* biofilms could be impacted by the complexity of the biofilm architecture, with increased turbidity resulting from detachment of biofilm cells. To further examine this possibility, we measured the optical density of buffer obtained after washing biofilm-coated membranes from the CBRs and recorded this value as relative detachment (Fig. 5). There was minimal detachment of parent cells from biofilms formed in the absence of antibiotics or in the presence of Gm. However, there was a significant increase in detached cells when the parent biofilm became more structurally complex after exposure to DAP. In addition, Δ*epaOX* biofilms were much less stable overall than parent biofilms in both the absence and presence of antibiotics. These data indicate that increased complexity in 3D architecture of the biofilm is associated with structural instability and increased susceptibility to antibiotics.

### Variation in lectin-binding specificity and polysaccharide composition of Δ*epaOX* is correlated with decreased biofilm integrity and antibiotic resistance

**Fig. 6:**
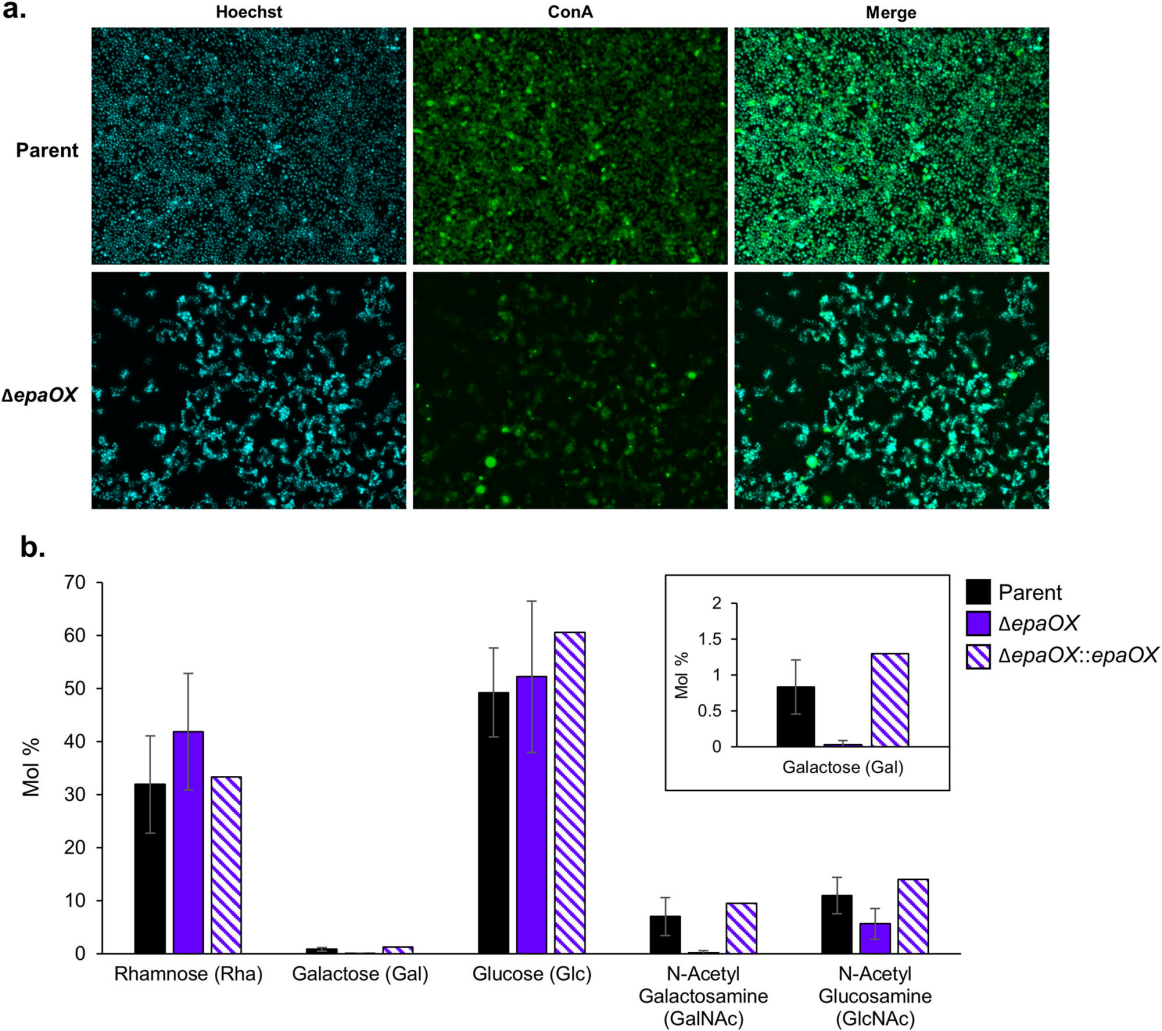
Deletion of *epaOX* affects lectin-binding ability and polysaccharide composition. **a** Immunofluorescence micrographs of parent and Δ*epaOX* biofilms. *E. faecalis* cells were cultured on Aclar membranes in CBRs for 24 h and stained with the DNA label Hoechst 33342 (*blue*) and the fluorescein-labeled lectin concanavalin A (ConA—*green*). Representative images at a magnification of 100× are shown from experiments performed in triplicate. **b** Carbohydrate composition of purified polysaccharides obtained from parent, Δ*epaOX*, and Δ*epaOX::epaOX* strains. *E. faecalis* strains were cultured to exponential phase for polysaccharide extraction. Error bars represent standard deviation (*n* = 3 for parent, Δ*epaOX*; *n* = 1 for Δ*epaOX::epaOX*)

We used lectin-binding assays and biochemical analysis to examine the correlation between the cell-wall polysaccharide composition of *E. faecalis* strains and the corresponding biofilm architecture and antibiotic resistance. Out of eight total fluorescein-labeled lectins that were screened, Concanavalin A (ConA) was the only lectin that efficiently bound and stained *E. faecalis* biofilm cells. ConA bound strongly to parent biofilm cells with distinct staining of the cell envelope. However, only a subset of Δ*epaOX* biofilm cells interacted with ConA and the staining pattern was diffuse compared to parent (Fig. 6a). To determine how Epa polysaccharides could affect ConA binding, we submitted purified polysaccharides from parent and Δ*epaOX* strains for glycosyl composition analysis using combined gas chromatography/mass spectrometry (GC/MS) by acid methanolysis (Complex Carbohydrate Research Center, University of Georgia). Similar to previous reports,^11, 20^ our results demonstrate that rhamnose and glucose are the main polysaccharides detected in OG1RF and that the minor components of Epa are galactose, GalNAc, and GlcNAc (Fig. 6b). Deletion of *epaOX* resulted in similar amounts of rhamnose and glucose compared to parent, virtually undetectable amounts of galactose and GalNAc, and GlcNAc levels that deviated among replicates. The preferred sugar specificities for ConA are α-linked mannose and α-linked d-glucose,^21^ which are either nonexistent (mannose) or similar in abundance (glucose) between both the strains. These data suggest that ConA is interacting with a non-preferred sugar substrate that is more accessible on the parent biofilm cells, or that biofilm architecture itself impacts binding. Therefore, the composition of Epa polymers may influence the interaction between antibiotics and *E. faecalis* biofilm cells impacting biofilm-associated antibiotic resistance. However, antibiotic sequestration experiments using purified polysaccharide from parent and Δ*epaOX* strains demonstrated no differential sequestration of DAP or Gm (Supplementary Fig. 4). These data instead suggest that antibiotic resistance is indirectly affected through Epa effects on biofilm architecture.

## Discussion

In some bacteria, elevated resistance to antibiotics in biofilms has been attributed to increased expression of genes whose products confer increased resistance. In *Pseudomonas aeruginosa*, enhanced expression of genes encoding diverse proteins including a DNA regulator, glucosyltransferase, efflux pump, type VI secretion component, putative two-component regulatory system, and hypothetical proteins have been linked to biofilm-induced resistance.^6, 22–26^ Similarly, *Escherichia coli* biofilms are rendered more resistant to antibiotics as a result of increased regulation of multi-drug efflux pumps and a putative protein that affects cell-wall composition.^27^ In this study, we determined that *E. faecalis epaI* and *epaOX* gene expression is not increased in biofilms, arguing against increased expression as the mechanism for *epa*-mediated biofilm antibiotic resistance. However, our data do not exclude the possibility of other *E. faecalis* biofilm-associated resistance mechanisms that involve altered gene expression.

Another potential mechanism for increased antibiotic resistance within biofilm cells is altered interaction of antibiotics with their target sites as a result of changes in the cell envelope. To begin to address this question, we screened a panel of fluorescein-labeled lectins for the ability to bind parent and Δ*epaOX* biofilm cells. Our data showed that the lectin ConA bound to parent, but not Δ*epaOX* biofilm cells. These data suggest that a Δ*epaOX* mutant could also exhibit altered interaction with antibiotics due to changes in surface properties, permeability, alteration of an antibiotic target, or differences in sequestration of antibiotic by polysaccharide. We showed previously altered polysaccharide production by the Δ*epaOX* mutant.^9^ Polysaccharide components of the biofilm matrix have been reported in *P. aeruginosa* and in the yeast *Candida albicans* to directly sequester antimicrobials, and these organisms also produce increased amounts of cyclic glucans within biofilm cells, which can bind antimicrobials to prevent access to their target sites.^6, 28^ Epa polysaccharides could also sequester antibiotics within the *E. faecalis* biofilms. However, we found that purified polysaccharides from parent and Δ*epaOX* strains did not differentially bind DAP or Gm arguing against antibiotic sequestration as the mechanism of *epa*-mediated biofilm-associated antibiotic resistance.

The GTF genes we analyzed are localized to the *epa* gene cluster, which includes the *epaA-R* genes^11, 12^ and additional downstream genes including *epaOX*.^9, 29^ There are seven predicted GTFs within the 18 annotated *epa* gene cluster and three additional predicted GTFs (including *epaOX*) in the OG1RF region immediately downstream of *epaR*.^29^ Previous reports have suggested that EpaB is a rhamnosyltransferase that helps construct the Epa backbone.^11^ Our data show that the Epa rhamnopolysaccharide was deficient in galactose and GalNAc upon deletion of *epaOX* suggesting a role in Epa decoration. The V583-associated gene *epaX* was also shown to help decorate Epa;^10^ however, we determined no functional redundancy to the OG1RF *epaOX* GTF (data not shown). These cumulative results have provided some functional characterization of the *epa* GTFs. Our current and future efforts will focus on the impact of antibiotics on Epa composition in biofilm cells. We have preliminary data demonstrating that the Epa content is similar between untreated and treated planktonic and biofilm cells (Supplementary Fig. 5). However, the exact composition of each monosaccharide may differ; therefore, we aim to perform large-scale cultivation experiments to obtain adequate quantities of polysaccharide for complete biochemical analysis.

The Epa polysaccharides are predicted to be buried within the *E. faecalis* cell wall.^20^ We and others have demonstrated that deletion or disruption of *epa* genes results in aberrations in cellular morphology.^9–11^ Since Epa appears integral in maintaining cell shape, we investigated its role in biofilm structure. Our data shows that deletion of *epaOX* markedly affects biofilm architecture. The Δ*epaOX* cells chain, clump, and form ultrastructured microcolonies compared to the monolayer biofilms formed by parent and the Δ*epaI* mutant. This drastic architectural change resulting from deletion of *epaOX* led us to hypothesize that biofilm architecture may play an important, intrinsic role in biofilm-associated antibiotic resistance.

To determine if biofilm architecture directly impacts antibiotic resistance, we analyzed biofilm development in the absence and presence of subinhibitory DAP and Gm concentrations using SEM. Surprisingly, it was the parent biofilm architecture that drastically changed after exposure to DAP or Gm, resembling Δ*epaOX* biofilm architecture in the absence of antibiotics, which remained relatively unchanged following antibiotic challenge. Parent cells formed a monolayer biofilm in the absence of antibiotics with sporadic microcolony formation only after 48 h of growth. However, upon exposure to subinhibitory concentrations of DAP parent biofilm cells rapidly reorganized into highly structured microcolonies reminiscent of Δ*epaOX* biofilms. Microcolony formation of parent cells in the presence of Gm was more subtle, but readily apparent (Supplementary Fig. 2).

This is not the first report demonstrating reorganization of bacterial biofilms. Moormeier et al.^30^ showed that *Staphylococcus aureus* biofilms change their matrix composition in order to transition from a multiplication stage to exodus. During the exodus stage, a subpopulation of biofilm cells detach and tower structures of robust growth develop at distinct foci.^30^ In *P. aeruginosa* multiple growth conditions and regulatory factors contribute to microcolony development;^31–35^ however, the extent to which microcolony formation results from rearrangement of monolayers is unclear.

The function of microcolonies in regard to environmental survival or virulence is likely to vary depending on the species and growth conditions. It has been proposed that *S. aureus* tower biofilms are important for pathogenesis enabling the bacteria to detach and disseminate to additional infection sites.^7, 36, 37^ In *P. aeruginosa*, the prototypical mushroom-shaped biofilm microcolonies are more resistant to detergent and antibiotics than less structured biofilms produced by certain mutants.^38, 39^ We showed that transition of *E. faecalis* biofilm structure from monolayers to microcolonies resulted in decreased stability and enhanced biofilm detachment. The Δ*epaOX* biofilm demonstrated the greatest extent of microcolony development and highest amount of cellular detachment in the presence and absence of antibiotics, while parent biofilms that restructured from monolayers to microcolonies in the presence of DAP also showed a high degree of cellular detachment. We have also shown that two independent clinical isolates (FVE12,^40^ MMH594^41^) exhibit increased relative detachment and cell death/permeability of biofilm cells after exposure to DAP suggesting antibiotic-induced biofilm instability is common among *E. faecalis* species (Supplementary Fig. 6). While increased biofilm detachment could be advantageous for bacterial dissemination during infection, detachment could be disadvantageous for *E. faecalis* by making the detached bacteria more susceptible to antibiotics or the immune response.

Microcolony formation in CBR grown *E. faecalis* biofilms could be viewed as a stress response where the source of stress for parent biofilms is antibiotics, while Δ*epaOX* mutant cells may be experiencing increased stress in the absence of antibiotics due to cell envelope defects. In support of this hypothesis, RT-qPCR analysis demonstrated that *E. faecalis* parent biofilms treated with DAP or Gm showed increased expression of three stress response genes (*sigV*, *croR*, and *liaR*) compared to untreated biofilms (Supplementary Table 2). These three stress response genes show increased expression in response to environmental pressure, cell-wall active antibiotics, and cell envelope damage,^42–45^ respectively, in *E. faecalis* and other organisms. Interestingly, Gm-exposed parent biofilms exhibited the largest fold-change increase in the stress response genes even though DAP-exposed parent biofilms demonstrated the most drastic biofilm architecture phenotypes. When expression of these three genes in biofilm cells of untreated parent and Δ*epaOX* was compared, there was a modest increase in *liaR* expression in Δ*epaOX* biofilms and little effect on *croR* or *sigV* transcription. However, it is possible the Δ*epaOX* mutant could be experiencing cell envelope stress during both planktonic and biofilm growth, complicating interpretation of analysis. We are currently developing an inducible gene-specific knockdown system that may enable induction of the Δ*epaOX* cell envelope defects during biofilm growth to better address this issue.

Interestingly, similar studies examining the transcriptome of antibiotic treated biofilms has been performed in *Klebsiella pneumoniae*. Van Laar et al.^46^ exposed preformed *K. pneumoniae* biofilms to sublethal, but not subinhibitory, concentrations of the carbapenem antibiotic imipenem and determined that there was differential regulation of the stress response with some genes being upregulated while others were downregulated. The authors also demonstrated that imipenem exposure resulted in a reversible morphological aberration in cell size, shape, and appearance of additional extracellular material, not unlike the phenotypes we have observed herein for *E. faecalis* antibiotic exposure. These complementary experimental approaches performed in two distinct organisms highlight the potentially congruent pathways employed by bacteria to sense and respond to environmental insult.

*E. faecalis* is an important pathogen in HAIs and understanding the mechanisms that increase antibiotic resistance to this recalcitrant microbe are of extreme importance. Our work provides insight into the role of Epa polysaccharides in biofilm architecture. In addition, our data identified conditions where *E. faecalis* biofilms with increased 3D complexity displayed decreased structural stability. Currently, our highest priority is to elucidate the mechanism of antibiotic-induced rearrangement from monolayer to microcolony biofilms. Our data suggests that this transition occurs by division and possibly by rearrangement of the adherent biofilm cells in the monolayers rather than by recruitment of new cells from the planktonic phase. We are initiating single cell analysis of adherent cells during the transition to address this issue. The current data also provide rationale for future experiments to determine the transcriptional response of *E. faecalis* biofilms to DAP and Gm on a genome-wide level. RNAseq-based identification of the most highly induced genes will enable design of genetic screens to identify the critical sensors and effectors of the changes in biofilm architecture induced by each antibiotic. Ultimately, a better understanding of the role of the cellular architecture of *E. faecalis* biofilms in antibiotic resistance may inform improved strategies to treat or prevent recalcitrant infections involving enterococcal biofilms.

## Materials and methods

### Bacterial strains and growth conditions

*E. faecalis* strains used in this study include the parent strain OG1RF,^47^ and isogenic derivatives Δ*epaOX* (JD102), Δ*epaI* (JD106), and Δ*epaOX::epaOX* (JD102[pJLD4]).^9^ *E. faecalis* strains were cultured overnight at 37 °C in brain heart infusion (BHI) medium (Becton, Dickinson and Co., Franklin Lakes, NJ) using static conditions. Tryptic soy broth without dextrose (TSB^-d^) (Becton, Dickinson and Co.) was used for all other experiments. Antibiotics were added to the medium as appropriate: DAP (Biotang Inc., Lexington, MA) at 6 µg/ml with 50 mg/l CaCl_2_, gentamicin at 37.5 µg/ml, spectinomycin at 1000 µg/ml, erythromycin at 10 µg/ml, or nisin at 25 ng/ml. All antibiotics were purchased from Sigma-Aldrich (St Louis, MO) unless stated otherwise. The number of replicates performed for each experiment are included in the figure legends and table footnotes.

### RNA purification and RT-qPCR

*E. faecalis* biofilm and planktonic cells were obtained from CBRs that were set-up as described below. To compare *epaI* and *epaOX* gene expression, polycarbonate coupons were removed aseptically after 24 h incubation and placed in six-well plates (four coupons/well) containing 5 ml distilled water and incubated for 5 min at room temperature to remove non-adherent cells. Aclar films were placed in 50 ml conical tubes (four Aclar films/tube) containing 30 ml KPBS and inverted to remove non-adherent cells. To obtain attached biofilm cells, twelve coupons and four Aclar were placed per 50 ml conical tube containing 8 ml KPBS supplemented with 2 mM EDTA and 16 ml of RNAprotect (Qiagen, Valencia, CA) and vortexed at 4 °C for 5 min. Vortexed biofilm cells were transferred to a new conical tube and centrifuged (6640×*g*) at 4 °C for 15 min for RNA extraction. Four milliliters of planktonic culture was removed from the reactor vessel and incubated with two volumes RNAprotect. After 5 min incubation at room temperature, planktonic cells were pelleted.

To compare stress response gene expression, Aclar films were removed after 8 h exposure to either DAP or Gm and washed as described above to remove non-adherent cells. To obtain attached biofilm cells, Aclar films were submerged in 4 ml KPBS supplemented with 2 mM EDTA and physically scraped off the film using one sterile razor blade per Aclar film. The scraped biofilm cell suspension was incubated with 8 ml RNAprotect for 5 min at room temperature and pelleted. One-hundred milliliters of planktonic culture was removed from the reactor vessel and pelleted. Insufficient quantities of planktonic culture were obtained from CBRs containing Gm; therefore, parent cells were cultured in batch using TSB^-d^ with or without 37.5 µg/ml Gm. Four-milliliters of planktonic culture was harvested at an OD_600_ of ~ 0.5 and incubated with 8 ml RNAprotect. After 5 min incubation at room temperature, planktonic cells were pelleted.

Biofilm and planktonic cells were lysed enzymatically using 30 mg/ml lysozyme and 500 U/ml mutanolysin in TE (10 mM Tris, pH8; 1 mM EDTA, pH8) for 10 min at 37 °C. Following lysis, RNA was extracted using the RNeasy mini kit (Qiagen) following the manufacturers protocol. One to four micrograms of total RNA was subjected to Turbo DNase treatment using the rigorous method as described (Ambion/ThermoScientific, Waltham, MA). Complementary DNA was prepared following the SuperScript III first-strand synthesis system for RT-PCR kit (Invitrogen/ThermoScientific) using random hexamers. Conventional PCR using universal 16 S primers was used to verify DNA removal. RT-qPCR was performed using SYBR green master mix and an iCycler iQ5 (Bio-rad, Hercules, CA). Total reaction volumes per well were 25 µl containing 200 nM of each primer. Primer pairs and melting temperature (Tm) used for RT-qPCR are found in Supplementary Table 1. Three-step RT-qPCR parameters were as follows: 3 min at 95 °C (one cycle); 10 s at 95 °C, 30 s at 53 °C or 59.5 °C, 30 s at 75 °C (40 cycles); 50°–95 °C melt curve with 0.5° temperature change. The primer efficiencies and cycle threshold (*C*t) measurements were calculated by the iQ5 Optical System Software version 2.1 (2009; Bio-Rad). All experiments met the following criteria: primer efficiency 78–100%; *r* ^2^ ≥ 0.995; and *C*t for no-RT control samples >35. The Pfaffl method was used to analyze data and calculate relative fold-change normalized to *relA* (Pfaffl 2001). RT-qPCR was performed on technical replicates from two biological samples of biofilm and planktonic cells.

### Polysaccharide purification and glycosyl composition analysis

*E. faecalis* parent, isogenic mutants, and complemented strains were cultured in 500 ml TSB^-d^ containing appropriate antibiotics, if necessary for plasmid maintenance, at 37 °C under static conditions to an OD_600_ of 0.4–0.6 (~ 1x10^8^ CFU/ml). Polysaccharides were extracted as described previously^9^ with modifications. Briefly, pelleted cells were washed with 10 ml of sucrose solution (25% sucrose, 10 mM Tris-HCl pH 8), resuspended in 15 ml sucrose solution supplemented with 1 mg/ml lysozyme and 10 U/ml mutanolysin and incubated with gentle agitation at 37 °C overnight. Supernatant fractions were collected from pelleted cells and treated with 200 µg/ml RNase A, 20 U/ml DNase, 5 mM MgCl_2_, and 1 mM CaCl_2_ at 37 °C for 8 h. Proteinase K was added at 50 µg/ml to the supernatant and incubated at 37 °C overnight. Remaining impurities were extracted using 500 µl chloroform. The aqueous phase was transferred to a new tube following centrifugation (6640×*g*) for 15 min. Polysaccharides were precipitated by adding ethanol to a final concentration of 75% and incubation at −70 °C for 30 min followed by 60 min centrifugation (6640×*g*) at 4 °C. Precipitated pellets were washed using 75% ethanol and allowed to air dry. Polysaccharide samples were submitted for glycosyl composition analysis to the Complex Carbohydrate Research Center (University of Georgia), which performed combined GC/MS using acidic methanolysis.

### Biofilm growth

Biofilms were cultured in CBRs on Aclar fluoropolymer film (Electron Microscopy Sciences, Hatfield, PA) and polycarbonate coupons (BioSurface Technologies Corp., Bozeman, MT). The CBR was assembled using 100% TSB^-d^ in the reactor vessel that contained eight Aclar films secured by polypropylene rods containing polycarbonate coupons. The vessel was placed on a stir plate rotating at 130 rpm and was attached via peristaltic pump tubing to a carboy containing 10% TSB^-d^ continuous flow medium. The entire CBR assembly was placed in a 37 °C room. Two-milliliters of overnight culture at a cell density of approximately 1 × 10^9^ CFU/ml was used to inoculate the CBR vessel. To allow bacterial attachment, the inoculated CBR was run for 4 h in batch culture at 130 rpm before starting the peristaltic pump at a flow rate of 8 ml/min for 20 h to enable a continuous flow of medium. After 24 h, the CBR carboy was exchanged for fresh 10% TSB^-d^ medium supplemented with DAP, gentamicin, or no antibiotics and incubated with a flow rate of 8 ml/min for 24 h. Samples were removed and rinsed in 30 ml KPBS at the following time points: 24 h (0 h antibiotic exposed), 32 h (8 h antibiotic exposed), and 48 h (24 h antibiotic exposed). Rinsed Aclar membranes were prepared for microscopy as described below. To estimate the relative detachment of biofilm cells, 1 ml from the 30 ml rinse suspension was measured at an OD_600_ at the designated time points.

### Immunofluorescence microscopy

Biofilms cultured on Aclar film were rinsed 3× using KPBS and stained for 15 min at room temperature with either 5 µg/ml Hoechst 33,342 and 20 ng/µl fluorescein-labeled Concanavalin A (Vector Laboratories, Burlingame, CA), or 2.5 µM Syto9 and 16 µM PI. Other lectins tested include fluorescein-labeled Soybean (SBA), wheat germ (weak interaction with parent and Δ*epaOX* [WGA]), *Dolichos biflorus* (DBA), *Ulex europaeus* (UEA 1), *Ricinus communis* (RCA_120_), Peanut (PNA), and Banana lectin (weak interaction with parent [BanLec]). All stains were purchased from Molecular Probes/ThermoFisher Scientific unless stated otherwise. Immunolabeled samples were either directly visualized by wet-mount or fixed in 2% paraformaldehyde in KPBS overnight at room temperature and mounted. Prolong diamond antifade mountant (ThermoFisher Scientific) was added to fixed Aclar on slides with a cover glass spacer to avoid damaging the biofilm structure (SecureSeal Adhesive, Electron Microscopy Sciences). Images were captured with a Zeiss AX10 using Zen 2.1 software as a wide-field snapshot or *z*-stack with a 20 × 0.8 numerical aperture (NA) or 100 × 1.3 NA objective. The images presented are maximum intensity projections obtained using the Fiji ImageJ package.^48^

### Scanning electron microscopy (SEM)

Reagents were purchased from Electron Microscopy Sciences (Hatfield, PA) unless otherwise noted. Samples were prepared using the cationic dye stabilization methods originally developed by Erlandsen et al.^49^ and modified as we have reported previously.^50^ Briefly, the Aclar films were rinsed 3× with KPBS and fixed (primary fixation) for 22 h in cacodylate-buffered solution (135 mM) containing methanol-free, EM-grade 2% formaldehyde and 2% glutaraldehyde, 4% sucrose, and 0.15% Alcian Blue 8GX (Sigma-Aldrich). Primary fixative was removed, Aclar coupons were rinsed 3× with sodium cacodylate buffer, and fixed (secondary fixation) for 1 h in a cacodylate-buffered solution (150 mM) containing 1% osmium tetroxide and 1.5% potassium ferrocyanide. Fixed samples were chemically dried using a graded ethanol series (25, 50, 70, 85, 95 [2x], 100% [2x]), processed in a CO_2_-based critical point dryer (tousimis, Rockville, MD), and sputter coated with a 1- to 2-nm layer of iridium (Leica EM ACE600, Buffalo Grove, IL). Low-voltage SEM imaging (0.8 kV) was performed using a Hitachi SU8230 field-emission instrument with the low-angle backscatter and secondary electron detectors.

### Data availability

All data that support the findings of this study are available from the corresponding author upon reasonable request.

## Electronic supplementary material

Supplemental Material

## References

[1] Sievert DM (2013). Antimicrobial-resistant pathogens associated with healthcare-associated infections: summary of data reported to the National healthcare safety network at the centers for disease control and prevention, 2009-2010. Infect. Control Hosp. Epidemiol..

[2] Hoyle BD, Costerton JW (1991). Bacterial resistance to antibiotics: the role of biofilms. Prog. Drug. Res..

[3] Potera C (1999). Forging a link between biofilms and disease. Science.

[4] Lewis K (2008). Multidrug tolerance of biofilms and persister cells. Curr. Top. Microbiol. Immunol..

[5] Mah TF, O’Toole GA (2001). Mechanisms of biofilm resistance to antimicrobial agents. Trends Microbiol..

[6] Mah TF (2003). A genetic basis for *Pseudomonas aeruginosa* biofilm antibiotic resistance. Nature.

[7] Donlan RM, Costerton JW (2002). Biofilms: survival mechanisms of clinically relevant microorganisms. Clin. Microbiol. Rev..

[8] Fernandez Guerrero ML, Goyenechea A, Verdejo C, Roblas RF, de Gorgolas M (2007). Enterococcal endocarditis on native and prosthetic valves: a review of clinical and prognostic factors with emphasis on hospital-acquired infections as a major determinant of outcome. Medicine (Baltimore)..

[9] Dale JL, Cagnazzo J, Phan CQ, Barnes AM, Dunny GM (2015). Multiple roles for *Enterococcus faecalis* glycosyltransferases in biofilm-associated antibiotic resistance, cell envelope integrity, and conjugative transfer. Antimicrob. Agents Chemother..

[10] Rigottier-Gois L (2015). The surface rhamnopolysaccharide Epa of *Enterococcus faecalis* is a key determinant of intestinal colonization. J. Infect. Dis..

[11] Teng F, Singh KV, Bourgogne A, Zeng J, Murray BE (2009). Further characterization of the *epa* gene cluster and Epa polysaccharides of *Enterococcus faecalis*. Infect. Immun..

[12] Xu Y, Murray BE, Weinstock GM (1998). A cluster of genes involved in polysaccharide biosynthesis from *Enterococcus faecalis* OG1RF. Infect. Immun..

[13] Singh KV, Lewis RJ, Murray BE (2009). Importance of the epa locus of *Enterococcus faecalis* OG1RF in a mouse model of ascending urinary tract infection. J. Infect. Dis..

[14] Xu Y, Singh KV, Qin X, Murray BE, Weinstock GM (2000). Analysis of a gene cluster of *Enterococcus faecalis* involved in polysaccharide biosynthesis. Infect. Immun..

[15] Ocvirk S (2015). Surface-associated lipoproteins link *Enterococcus faecalis* virulence to colitogenic activity in IL-10-deficient mice independent of their expression levels. PLoS Pathog..

[16] Hoff JS, Kristich CJ (2016). Thymidylate limitation potentiates cephalosporin activity toward Enterococci via an Exopolysaccharide-based mechanism. ACS Chem. Biol..

[17] Cotroneo N, Harris R, Perlmutter N, Beveridge T, Silverman JA (2008). Daptomycin exerts bactericidal activity without lysis of *Staphylococcus aureus*. Antimicrob. Agents Chemother..

[18] Pogliano J, Pogliano N, Silverman JA (2012). Daptomycin-mediated reorganization of membrane architecture causes mislocalization of essential cell division proteins. J. Bacteriol..

[19] Tran TT (2013). Daptomycin-resistant *Enterococcus faecalis* diverts the antibiotic molecule from the division septum and remodels cell membrane phospholipids. mBio.

[20] Hancock LE, Gilmore MS (2002). The capsular polysaccharide of *Enterococcus faecalis* and its relationship to other polysaccharides in the cell wall. Proc. Natl Acad. Sci. USA.

[21] Laboratories, V. Table of Lectin Properties. http://docs.vectorlabs.com/protocols/K4-K7.pdf.

[22] Beaudoin T, Zhang L, Hinz AJ, Parr CJ, Mah TF (2012). The biofilm-specific antibiotic resistance gene *ndvB* is important for expression of ethanol oxidation genes in *Pseudomonas aeruginosa* biofilms. J. Bacteriol..

[23] Whiteley M (2001). Gene expression in *Pseudomonas aeruginosa* biofilms. Nature.

[24] Zhang L (2013). Identification of genes involved in *Pseudomonas aeruginosa* biofilm-specific resistance to antibiotics. PLoS ONE..

[25] Zhang L, Hinz AJ, Nadeau JP, Mah TF (2011). *Pseudomonas aeruginosa tssC1* links type VI secretion and biofilm-specific antibiotic resistance. J. Bacteriol..

[26] Zhang L, Mah TF (2008). Involvement of a novel efflux system in biofilm-specific resistance to antibiotics. J. Bacteriol..

[27] Lynch SV (2007). Role of the *rapA* gene in controlling antibiotic resistance of *Escherichia coli* biofilms. Antimicrob. Agents Chemother..

[28] Nett J (2007). Putative role of beta-1,3 glucans in *Candida albicans* biofilm resistance. Antimicrob. Agents Chemother..

[29] Palmer KL (2012). Comparative genomics of enterococci: variation in *Enterococcus faecalis*, clade structure in *E. faecium*, and defining characteristics of *E. gallinarum* and *E. casseliflavus*. mBio.

[30] Moormeier DE, Bose JL, Horswill AR, Bayles KW (2014). Temporal and stochastic control of *Staphylococcus aureus* biofilm development. mBio.

[31] Banin E, Vasil ML, Greenberg EP (2005). Iron and *Pseudomonas aeruginosa* biofilm formation. Proc. Natl Acad. Sci. USA.

[32] O’Toole GA, Gibbs KA, Hager PW, Phibbs PV, Kolter R (2000). The global carbon metabolism regulator Crc is a component of a signal transduction pathway required for biofilm development by *Pseudomonas aeruginosa*. J. Bacteriol..

[33] Petrova OE, Sauer K (2009). A novel signaling network essential for regulating *Pseudomonas aeruginosa* biofilm development. PLoS Pathog..

[34] Petrova OE, Schurr JR, Schurr MJ, Sauer K (2012). Microcolony formation by the opportunistic pathogen *Pseudomonas aeruginosa* requires pyruvate and pyruvate fermentation. Mol. Microbiol..

[35] Yang L (2007). Effects of iron on DNA release and biofilm development by *Pseudomonas aeruginosa*. Microbiology.

[36] Fernandez Guerrero ML, Alvarez B, Manzarbeitia F, Renedo G (2012). Infective endocarditis at autopsy: a review of pathologic manifestations and clinical correlates. Medicine (Baltimore)..

[37] Thomas VC (2014). A central role for carbon-overflow pathways in the modulation of bacterial cell death. PLoS Pathog..

[38] Davies DG (1998). The involvement of cell-to-cell signals in the development of a bacterial biofilm. Science.

[39] Hentzer M (2001). Alginate overproduction affects *Pseudomonas aeruginosa* biofilm structure and function. J. Bacteriol..

[40] Leuck AM, Johnson JR, Dunny GM (2014). A widely used *in vitro* biofilm assay has questionable clinical significance for enterococcal endocarditis. PLoS ONE.

[41] Huycke MM, Spiegel CA, Gilmore MS (1991). Bacteremia caused by hemolytic, high-level gentamicin-resistant *Enterococcus faecalis*. Antimicrob. Agents Chemother..

[42] Benachour A (2005). The *Enterococcus faecalis sigV* protein is an extracytoplasmic function sigma factor contributing to survival following heat, acid, and ethanol treatments. J. Bacteriol..

[43] Comenge Y (2003). The CroRS two-component regulatory system is required for intrinsic beta-lactam resistance in *Enterococcus faecalis*. J. Bacteriol..

[44] Poole K (2012). Bacterial stress responses as determinants of antimicrobial resistance. J. Antimicrob. Chemother..

[45] Suntharalingam P, Senadheera MD, Mair RW, Levesque CM, Cvitkovitch DG (2009). The LiaFSR system regulates the cell envelope stress response in *Streptococcus mutans*. J. Bacteriol..

[46] Van Laar TA, Chen T, You T, Leung KP (2015). Sublethal concentrations of carbapenems alter cell morphology and genomic expression of *Klebsiella pneumoniae* biofilms. Antimicrob. Agents Chemother..

[47] Dunny GM, Brown BL, Clewell DB (1978). Induced cell aggregation and mating in *Streptococcus faecalis*: evidence for a bacterial sex pheromone. Proc. Natl Acad. Sci. USA.

[48] Schindelin J (2012). Fiji: an open-source platform for biological-image analysis. Nat. Methods.

[49] Erlandsen, S. L., Kristich, C. J., Dunny, G. M. & Wells, C. L. High-resolution visualization of the microbial glycocalyx with low-voltage scanning electron microscopy: dependence on cationic dyes. *The journal of histochemistry and cytochemistry: official journal of the Histochemistry Society* **52**, 1427–1435 (2004). 10.1369/jhc.4A6428.2004 PMC3957825 15505337

[50] Barnes, A. M., Ballering, K. S., Leibman, R. S., Wells, C. L. & Dunny, G. M. Enterococcus faecalis produces abundant extracellular structures containing DNA in the absence of cell lysis during early biofilm formation. *mBio*. **3**, e00193–00112 (2012). 10.1128/mBio.00193-12 PMC3413405 22829679

