# Supplementary material for: Restructuring of Enterococcus faecalis biofilm architecture in response to antibiotic-induced stress
Source: NPJ Biofilms Microbiomes. 2017 Jun 30;3:15. doi: 10.1038/s41522-017-0023-4 (PMC5493694; doi:10.1038/s41522-017-0023-4)
Supplement: Supplementary file 1 — Supplemental Material [file 41522_2017_23_MOESM1_ESM.pdf]

Supplementary Figure 1

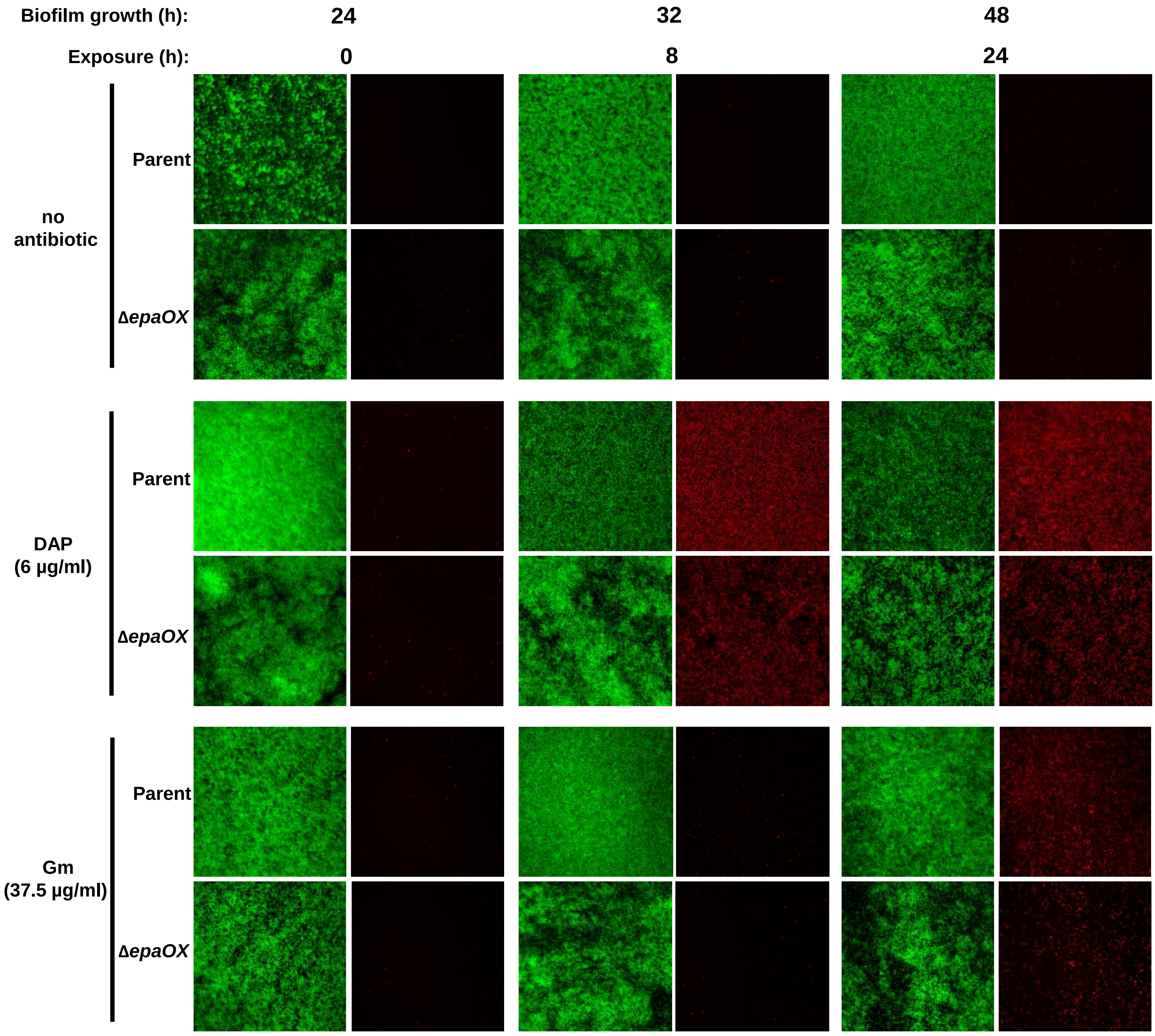

Supplementary Figure 2

a.

Gentamicin (37.5 µg/ml) effect on pre-formed biofilms

**Biofilm growth (h):**	24	32	48
**Gm exposure (h):**	0	8	24

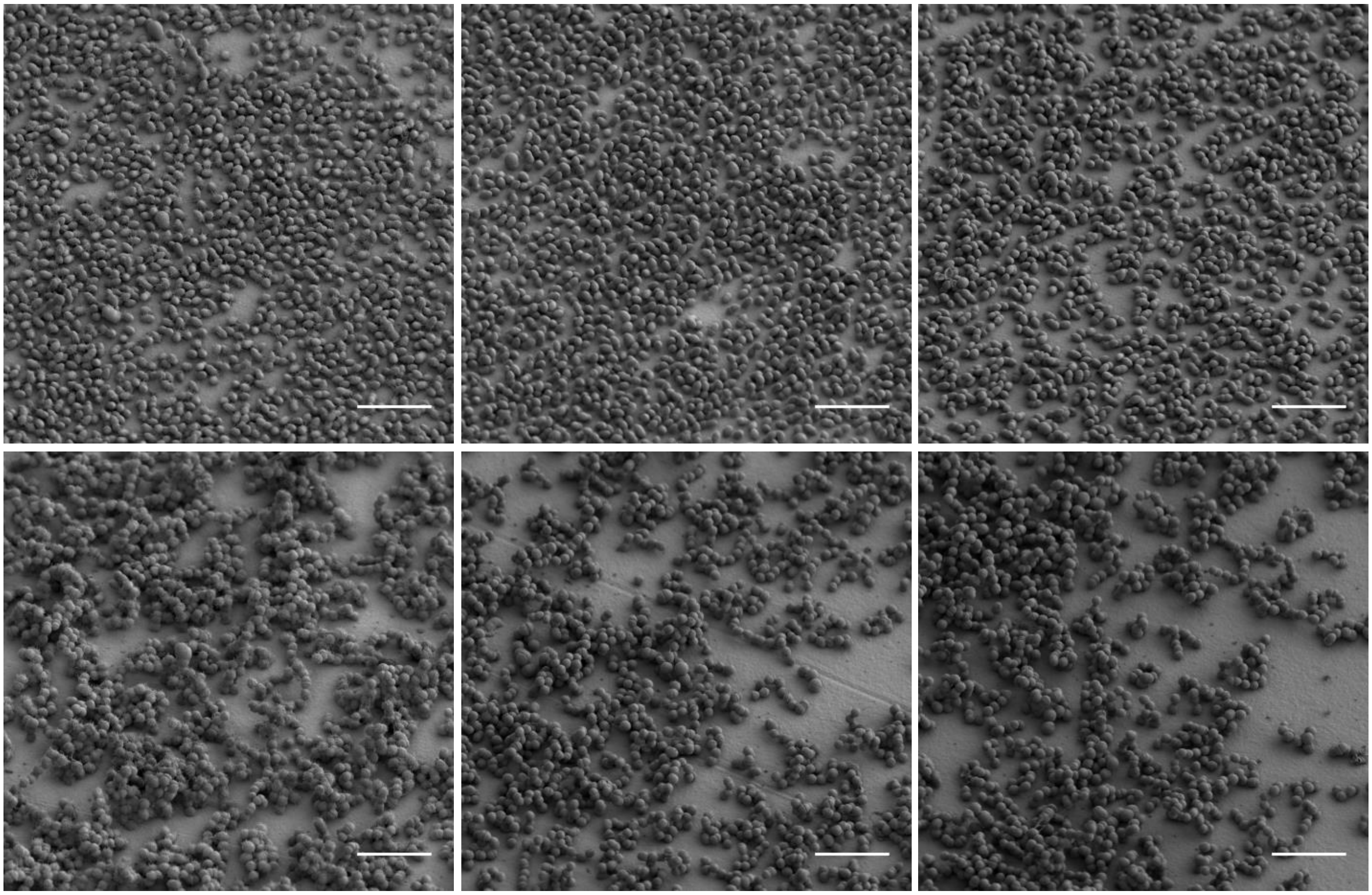

b.

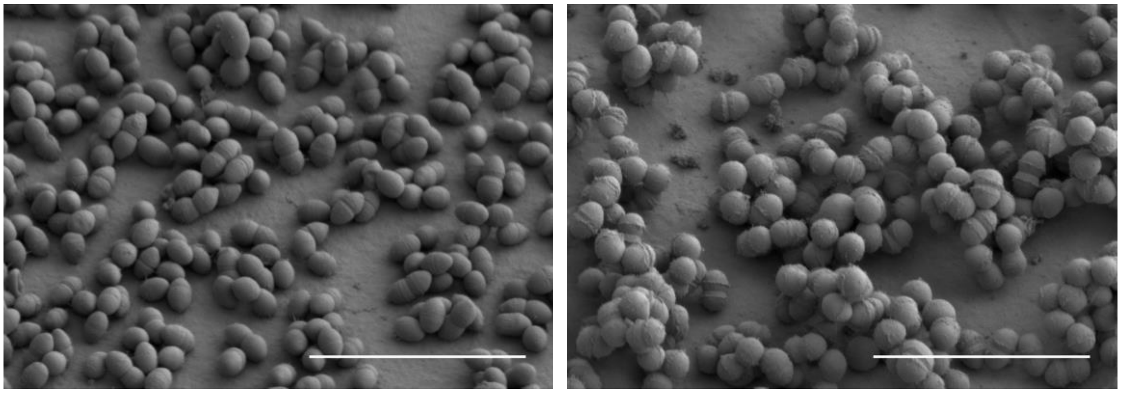

Supplementary Figure 3

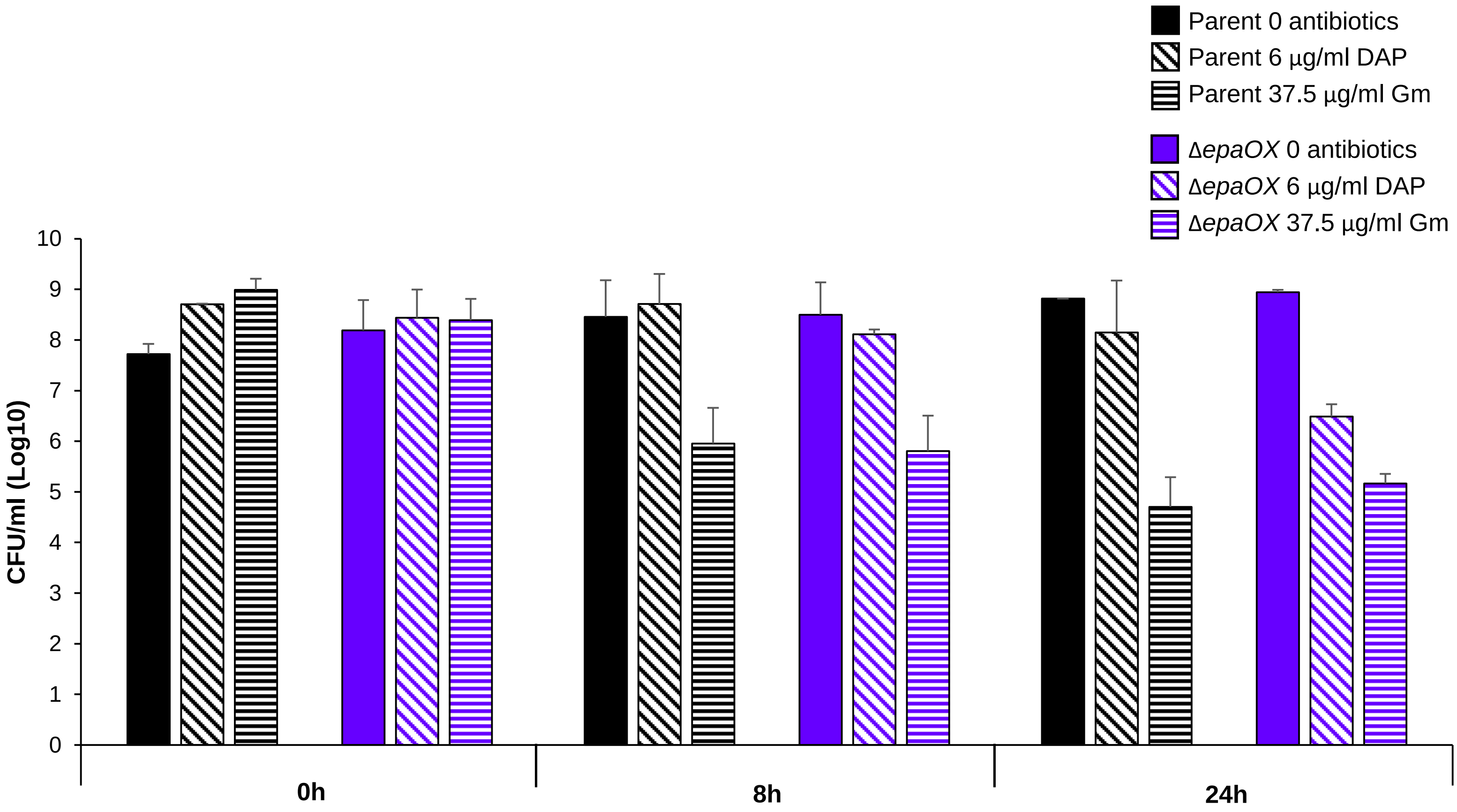

Supplementary Figure 4

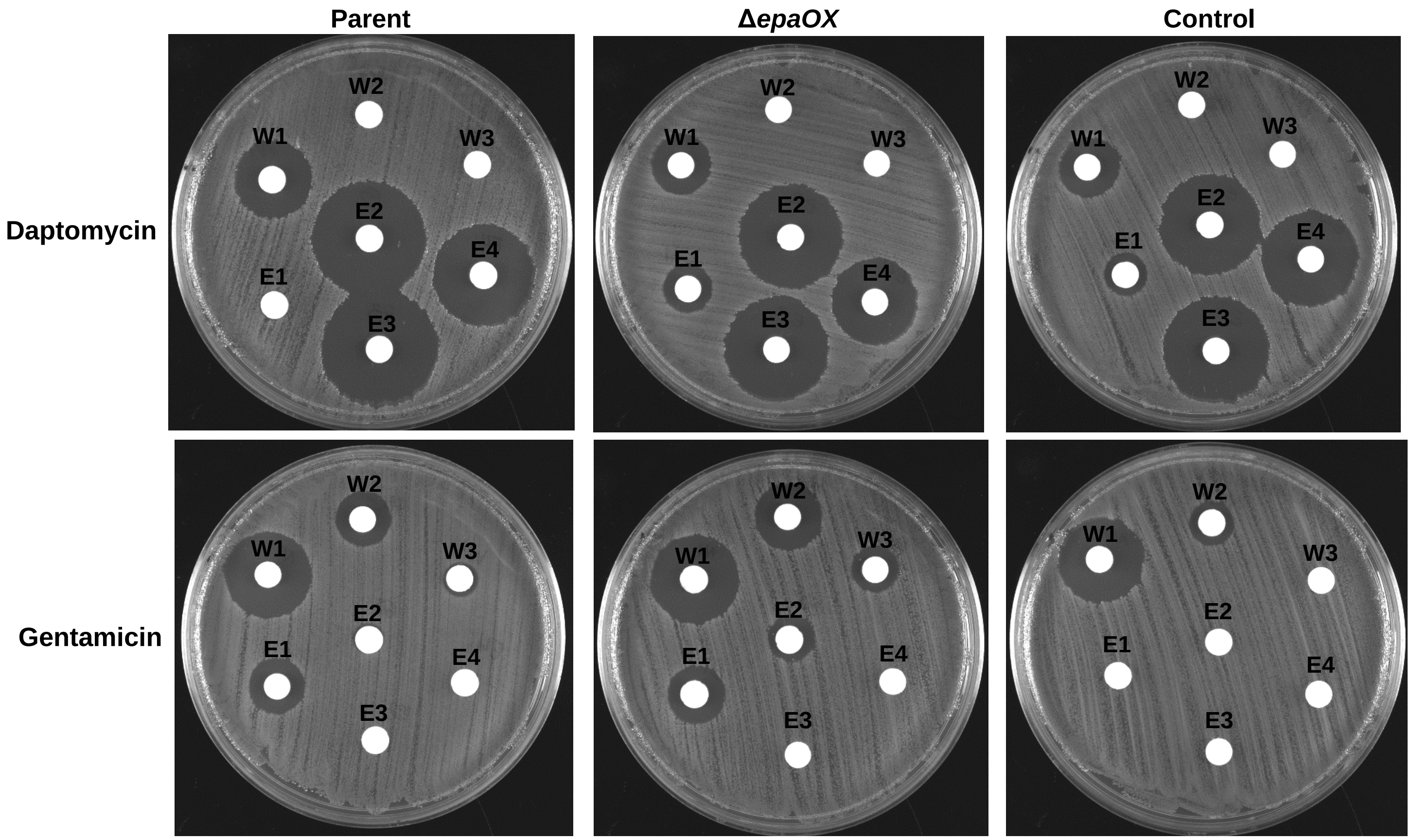

Supplementary Figure 5

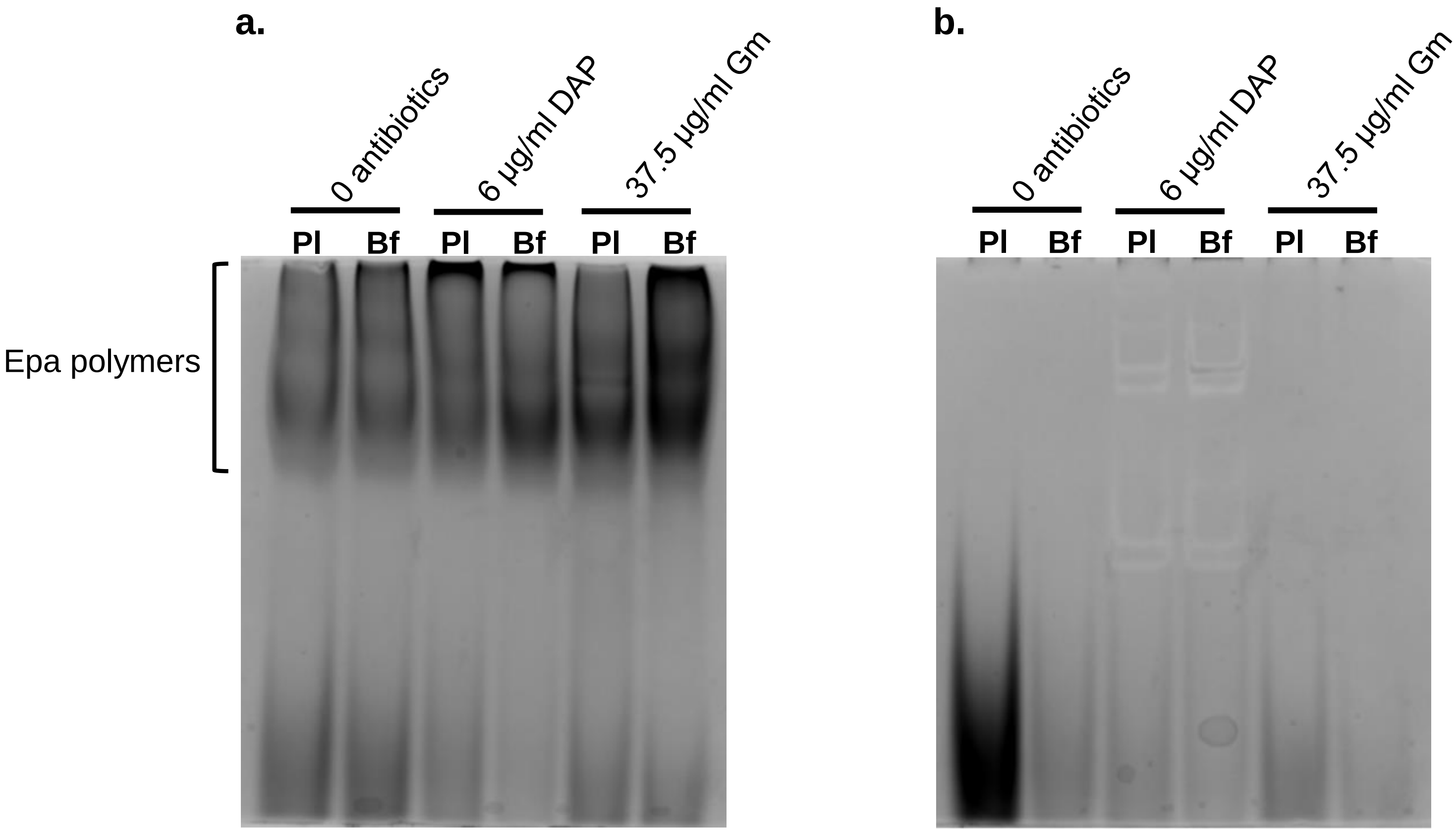

Supplementary Figure 6

a.

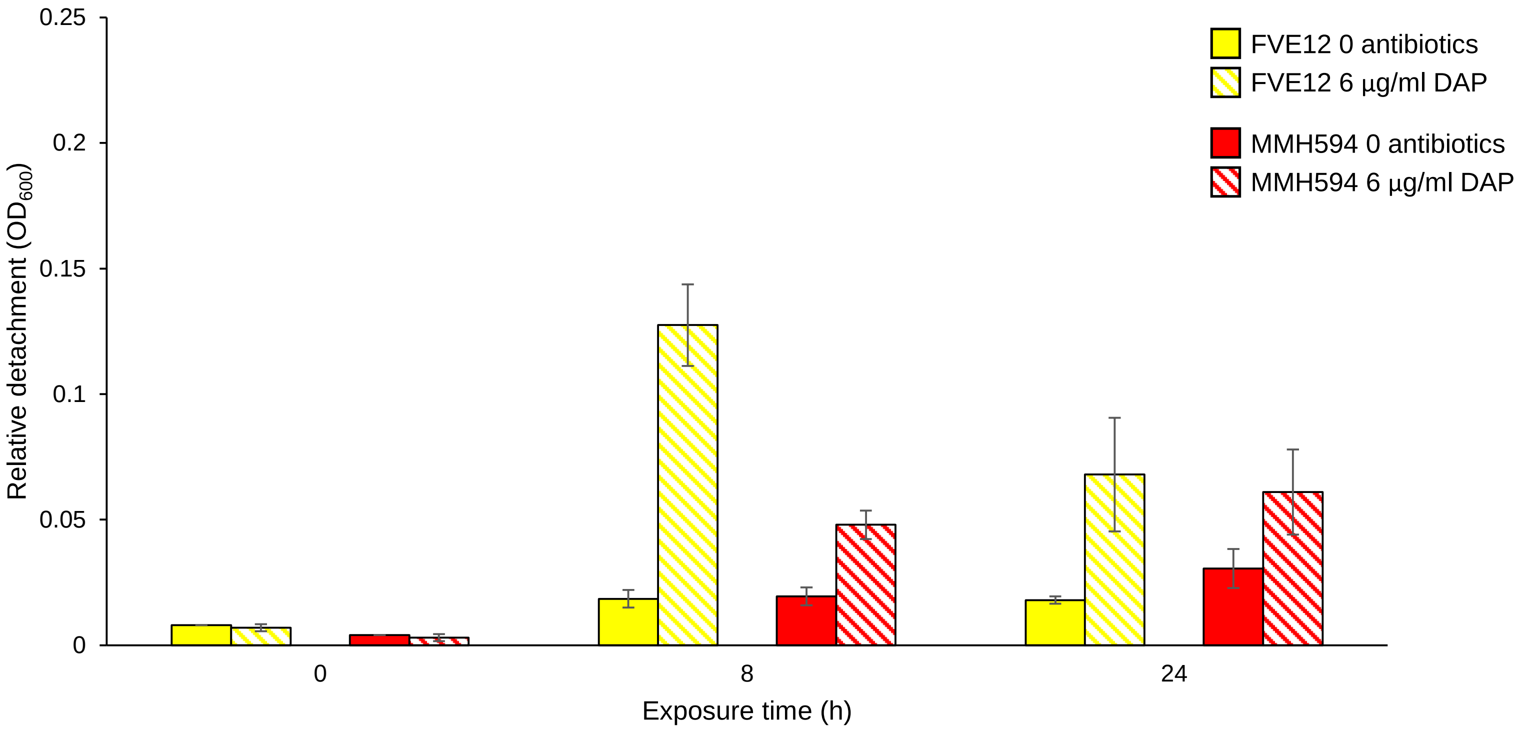

b.

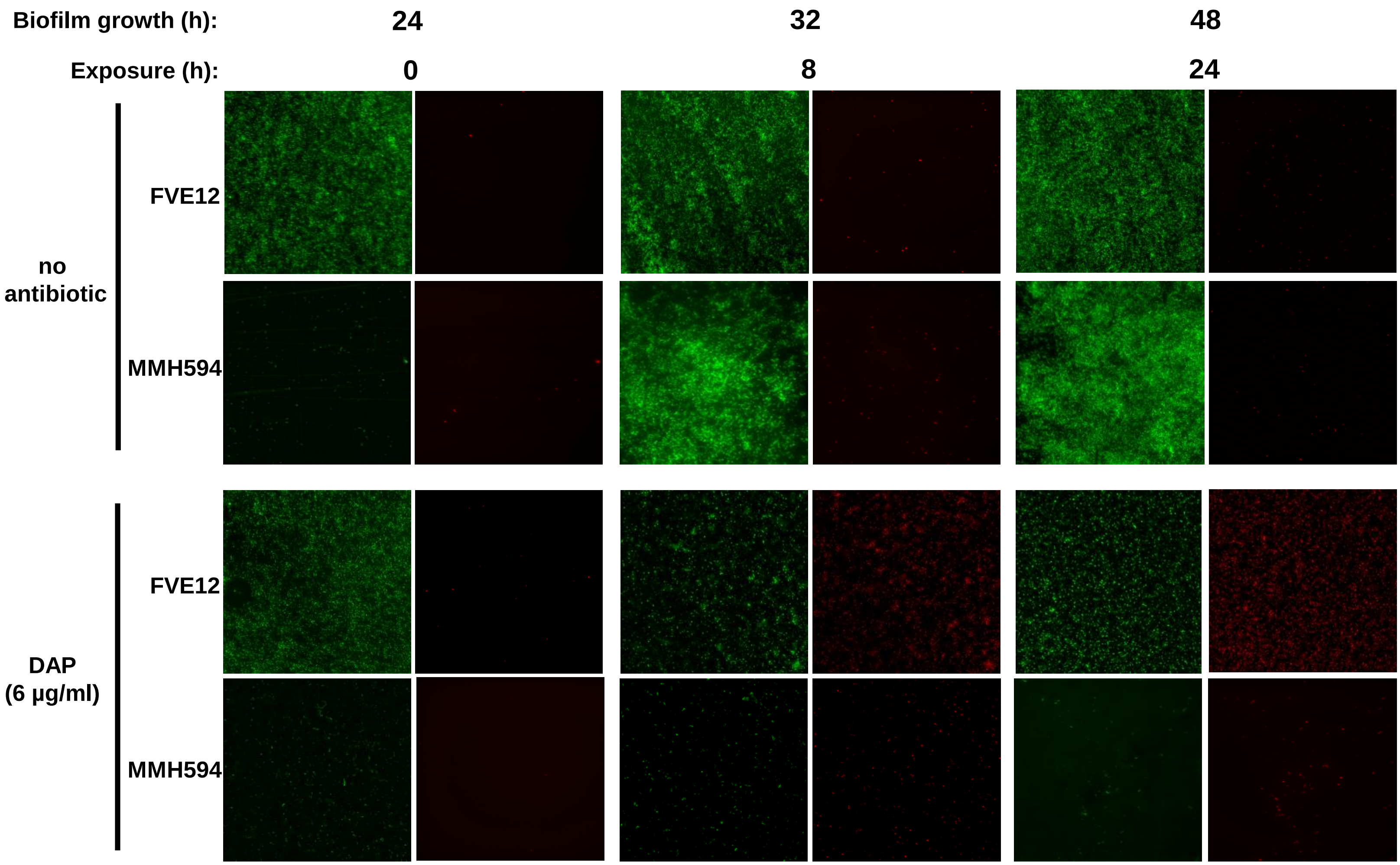

Supplementary Table 1. RT-qPCR primers used in this study

Name	Sequence (5' to 3')	Gene	Tm	Length (bp)
JD456	GGCTCGTTTCATAACCTC	*epaOX* (*OG1RF_11715*)	53°C	143
JD457	TCCGCTCTCTCTTTGTTG	*epaOX* (*OG1RF_11715*)	53°C	143
JD458	CCCTCTGCTAATGGTTCCAG	*epaI* (*OG1RF_11730*)	53°C	86
JD459	GAGTATGATGTGGCGGTTCA	*epaI* (*OG1RF_11730*)	53°C	86
JD464	CGACTTGCTTCGTCAGTTCA	*relA* (*OG1RF_11636*)	53° or 59.5°C	106
JD465	ACCCATGAGATGCACCAAA	*relA* (*OG1RF_11636*)	53° or 59.5°C	106
JD472	GAACCACGCTTCAAAATCCT	*sigV* (*OG1RF_12448*)	59.5°C	95
JD473	AAGAAGATGCCTTGGATGTGA	*sigV* (*OG1RF_12448*)	59.5°C	95
JD474	GAAGTAATGGCTCGGGTGAA	*croR* (*OG1RF_12535*)	59.5°C	85
JD475	GGCCGACTTCTAATTCATCG	*croR* (*OG1RF_12535*)	59.5°C	85
JD476	CATCATCAATAAAACTCGTCACAA	*liaR* (*OG1RF_12211*)	59.5°C	71
JD477	TTCAACAAAAGCGATCTTGAAA	*liaR* (*OG1RF_12211*)	59.5°C	71

Supplementary Table 2. Expression of stress response genes in biofilms (fold-change relative to *relA*) using RT-qPCR

	**DAP treated / untreated[1]**	**Gm treated / untreated[1]**	**Δ*epaOX* / OG1RF[2]**
liaR	5.2 ± 1.76	10.4 ± 1.06	2.0 ± 0.29
croR	6.9 ± 1.38	2.2 ± 0.83	0.9 ± 0.14
sigV	3.8 ± 2.65	25.9 ± 10.61	1.7 ± 1.22

[1]OG1RF daptomycin (DAP; 6 µg/ml, 50 mg/L $CaCl_2$) or gentamicin (Gm; 37.5 µg/ml) treated compared to untreated biofilm cells. Biofilms were cultured on Aclar fluoropolymer film for 24h prior to 8h of DAP, Gm, or mock treatment.
[2]Δ*epaOX* compared to OG1RF biofilm cells cultured on Aclar fluoropolymer film for 32h.
All experiments were performed in biological duplicate with technical replicates.

Supplementary Figure 1. *E. faecalis* biofilm permeability/viability in the absence and presence of antibiotics. Immunofluorescence microscopy was performed for live/dead biofilm staining using Syto9 (green - live) and propidium iodide (red - dead / more permeable) at a magnification of 20x. Representative images are shown from experiments performed in triplicate with similar results.

Supplementary Figure 2. Gentamicin effects on pre-formed *E. faecalis* biofilms. Experiments were performed as described for Figure 3, except Gentamicin (Gm), rather than DAP, was added to 24h pre-formed biofilms at a concentration of 37.5 µg/ml and the biofilms were examined using SEM after 8 and 24h of antibiotic exposure. (a) Gm effects on *E. faecalis* parent and Δ*epaOX* biofilms (Bars, 5 µm). (b) Higher magnification images demonstrating parent biofilm restructuring into microcolonies, and the loss of extracellular appendages on both strains. Bars, 5µm. Representative images are shown from experiments performed in triplicate with similar results.

Supplementary Figure 3. *E. faecalis* biofilm susceptibility to antibiotics. Quantitative counts of biofilm cells that were attached to Aclar membranes. Error bars represent standard deviation of biological replicates.

Supplementary Figure 4. Antibiotics are not differentially sequestered by purified parent and Δ*epaOX* polysaccharides. Disk diffusion assays depicting antibiotic retention to C18 columns pre-loaded with purified polysaccharide from parent or Δ*epaOX* strains. Control plates were not pre-loaded with polysaccharides. Eluates from the loaded and non-loaded columns were absorbed onto sterile disks and placed on to a lawn of daptomycin- and gentamicin- sensitive *Lactococcus lactis*. Zones of inhibition around disks absorbed with eluate fractions would be

indicative of antibiotic retention. W1-3, water washes; E1, 25% acetonitrile; E2, 50% acetonitrile; E3, 75% acetonitrile; E4, 100% acetonitrile. The data show zones of inhibition in all DAP eluate fractions of parent and Δ*epaOX* polysaccharide-loaded columns; however, the negative control exhibits the same inhibition pattern suggesting no sequestration of DAP. Both parent and Δ*epaOX* polysaccharides appear to sequester Gm. In total, these data indicate that purified parent and Δ*epaOX* polysaccharide do not differentially sequester DAP or Gm. Representative images are shown from experiments performed in triplicate with similar results.

Supplementary Figure 5. Effects of antibiotic exposure on polysaccharide content. Purified polysaccharides from (a) parent and (b) Δ*epaOX* planktonic (Pl) and biofilm (Bf) cells were obtained from CDC biofilm reactor cultures after 24h exposure to daptomycin (6 μg/ml DAP; 50 mg/L $CaCl_2$) or gentamicin (37.5 μg/ml Gm) and were electrophoresed through a 10% native polyacrylamide gel. Polysaccharide from Gm-exposed planktonic cells was obtained from batch culture experiments as described in Material and Methods. Polysaccharide content was detected using Stains-all. Representative images are shown from experiments performed in duplicate with similar results.

Supplementary Figure 6. Biofilm stability and permeability/viability of clinical *E. faecalis* strains FVE12 and MMH594 cultured in the absence and presence of antibiotics. (a) Relative detachment of biofilms cells from Aclar membranes after washing, as determined by measuring the OD_{600} of the wash buffer as described in the methods. Error bars represent standard deviation of biological replicates. (b) Immunofluorescence microscopy was performed for live/dead biofilm staining using Syto9 (green - live) and propidium iodide (red - dead / more permeable) at a magnification of 20x. Representative images are shown from experiments performed in duplicate with similar results.
